# Effect of a Personalized Mobile App on Glucose Control in Adults With Prediabetes and Type 2 Diabetes: Exploratory Pilot Randomized Controlled Trial

**DOI:** 10.2196/87692

**Published:** 2026-06-24

**Authors:** Elena Lalama, Marta Csanalosi, Stefan Kabisch, Saskia Wilson-Barnes, Lazaros P Gymnopoulos, Kosmas Dimitropoulos, Konstantinos Rouskas, Argiriou Anagnostis, Ioannis Oikonomidis, Leontios Hadjileontiadis, Veronique Cornelissen, Maria Hassapidou, Ioannis Pagkalos, Sofia Balula Dias, Kathryn Hart, Andreas F H Pfeiffer

**Affiliations:** 1 Department of Endocrinology and Metabolic Diseases Charité - Universitätsmedizin Berlin Berlin, Berlin Germany; 2 German Center for Diabetes Research Neuherberg, Bavaria Germany; 3 School of Biosciences, Discipline: Nutrition, Food and Exercise Science University of Surrey Faculty of Health and Medical Sciences Surrey United Kingdom; 4 Information Technologies Institute Centre for Research and Technology Hellas Thessaloniki Greece; 5 Institute of Applied Biosciences Centre for Research & Technology Hellas Thessaloniki Greece; 6 INTRASOFT International (Greece) Athens, Attica Greece; 7 Department of Electrical and Computer Engineering Aristotle University of Thessaloniki Thessaloniki Greece; 8 Department of Rehabilitation Sciences KU Leuven Leuven, Flanders Belgium; 9 Department of Nutritional Sciences and Dietetics International Hellenic University Thessaloniki Greece; 10 Faculdade de Motricidade Humana University of Lisboa Lisbon, Lisbon Portugal

**Keywords:** type 2 diabetes, personalized nutrition, glucose management, time in range, CGM, continuous glucose monitoring

## Abstract

**Background:**

The incidence of type 2 diabetes (T2D) continues to increase, and the lack of individualized therapy strategies hinders patient engagement with and commitment to a healthy lifestyle. The PROTEIN project aimed to facilitate users in choosing healthy living, thereby improving their metabolism and T2D management.

**Objective:**

This study aims to assess the efficacy of a personalized mobile app to achieve a 5% time in range (TIR) improvement over a 12-week intervention in adults with prediabetes or T2D.

**Methods:**

We conducted an exploratory pilot randomized controlled trial with 21 individuals with T2D or prediabetes who used a continuous glucose monitoring system and the PROTEIN mobile app for personalized meals and exercise recommendations based on their glucose levels and physical activity.

**Results:**

The TIR of the participants increased (*P*<.05; from 71.8%, SD 27.3% to 76%, SD 28.1%) with individual use of the PROTEIN app but did not achieve a 5% improvement overall; however, given the exploratory design and small sample size, this finding should be interpreted with caution. Glycated hemoglobin, fasting blood glucose, and body weight did not fluctuate throughout the 12-week intervention. The dropout rate was high, and the average duration of use of the PROTEIN app was 42 (range 5-84) days.

**Conclusions:**

Our results showed a modest increase in TIR with the use of the PROTEIN app; however, considering the exploratory design and small sample size, this finding should be interpreted as preliminary. Integrating wearables and automated personalization for well-being is an innovative approach that must keep pace with the accelerated development of ever-evolving technologies. The COVID-19 pandemic was a major obstacle to recruitment in our clinical trial.

**Trial Registration:**

ClinicalTrials.gov NCT05951140; https://clinicaltrials.gov/study/NCT05951140

## Introduction

Identifying optimal diets for individuals with type 2 diabetes (T2D) and prediabetes remains challenging [1]. The World Health Organization defines mobile health (mHealth) as medical and public health practices supported by mobile devices, which have become increasingly prevalent [2]. Together, these developments highlight the growing potential of digital tools to support personalized diabetes management.

Advances in artificial intelligence (AI) now enable the processing of large datasets to deliver personalized nutrition advice that aligns with individual values and preferences, potentially supporting sustained patient engagement in healthy dietary choices [3,4]. However, despite the constant and rapid evolution of diabetes-related mobile apps, few offer adaptive, evidence-based nutrition guidance for patients with T2D in Germany [5-7].

Given the significant interindividual variability in postprandial glucose responses, generic recommendations are insufficient. Integrating data from continuous glucose monitoring (CGM) and user preferences allows for more tailored interventions, using algorithms powered by machine learning and AI to enhance diabetes self-management [6,8-11].

Within the European H2020 “PROTEIN: PeRsOnalized nutriTion for hEalthy living” project (2019-2022), we aimed to develop and evaluate a personalized nutrition and exercise mobile app to support daily T2D management [12]. The PROTEIN project involved 20 partners from 11 European countries, focusing on health promotion through multidisciplinary collaboration. Our exploratory pilot study specifically assessed whether the PROTEIN app could improve dietary compliance and glucose management in T2D, using time in range (TIR) as the primary outcome - a metric not commonly used as a primary endpoint in similar randomized controlled trials (RCTs) [7]. TIR is the proportion of time that a person spends within a target range of glucose levels, usually between 70 and 180 mg/dL, as predetermined by a health practitioner or the person with diabetes [13].

Following Germany’s 2019 digital health reform, only approved apps can be prescribed for diabetes management, ensuring patient safety and reliability [14]. Only 6 of 59 mHealth apps have been approved for people with diabetes [15]. The PROTEIN trial provides prospective, controlled, long-term evidence on AI-driven mobile apps that deliver personalized recommendations using CGM data, user interaction, and preferences. This adaptive AI approach addressing engagement and patient engagement challenges is novel in digital therapeutics.

To our knowledge, this adaptive approach has not been reported in previous studies [16-19]. Therefore, our exploratory pilot study aimed to automatically customize meal plans and physical activity (PA) according to user phenotype and food preferences by integrating CGM data of patients with T2D. The primary objective was to increase the TIR of the study participants by 5% while improving eating behaviors.

Expanding upon existing research findings, we aimed to address the following questions:

1. Does TIR increase when using the PROTEIN app?
2. Are there associated changes in weight?

## Methods

### Study Design

We conducted a 12-week RCT in Germany (June-December 2022) to evaluate the PROTEIN app’s impact on glucose management in T2D.

#### The PROTEIN System

Developed and tested [20-24] by a European consortium, the PROTEIN system comprised:

1. Nutrium dashboard: used by nutritionists to manage participants and create meal plans using country-specific food composition datasets [25].
2. PROTEIN app: enabled participants to view, plan, and log meals and activities, and interact with personalized recommendations.
3. User dashboard: provided participants with an alternative visualization tool through a web browser.

The AI-driven advisor included a food and activity recommender system, a reasoning-based decision support system [8] built on the Nutrition & Activity Ontology for Healthy Living, which encoded the rules and relations used by the AI advisor [26], and generated individualized meal and activity plans every two weeks. These were based on the user profile, preferences, CGM data, and aligned with the “knowledge tracker,” a framework for the meal and PA suggestions [27] that was integrated into the application. Nutrition requirements followed European guidelines [28].

#### Intervention

We conducted a 12-week intervention. The participants used the PROTEIN app and the CGM Freestyle Libre (Abbott Diabetes Care, Wiesbaden, Germany) to collect data to enable further personalization. We randomized and allocated the participants into two groups after successful screening:

1. Start-group: participants used the activity tracker, CGM, and PROTEIN app for 12 weeks, followed by a 6-week period without the PROTEIN app, but where the wearables continued to be used.
2. Wait-group: participants only used the activity tracker and CGM for 6 weeks, followed by a 12-week period of using the PROTEIN app as well.

**Figure 1:**
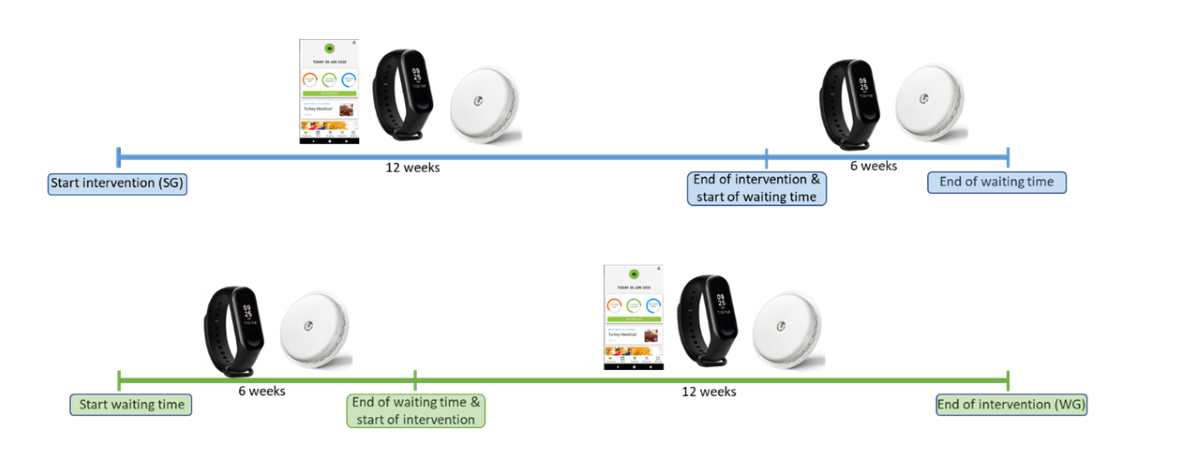
Study design illustrating the start-group (blue, top panel) and the wait-group (green, bottom panel).

This study design, as shown in Figure 1, allowed us to clearly understand the influence of the PROTEIN app. The randomization sequence was computer-generated (Microsoft Excel 2019, Microsoft Corporation) and was performed on-site by the study team. Stratification considered sex, age (cut-off 60 years), and BMI (cut-off 30kg/m^2^) at screening. Given the nature of the trial, blinding was not possible for either participants or study personnel. After generating the randomization sequence, participants were assigned on-site by the study team, without formal allocation concealment.

The participants allocated to the start-group attended their first clinical visit, where baseline clinical and laboratory assessments, including blood count, lipid panel, liver profile, uric acid, C-reactive protein, fasting glucose, hemoglobin A_1c_ (HbA_1c_), ankle-brachial index, and body composition, were collected after an overnight fast.

On a typical day, the participants would open the app to review the recommended meal and activity plans, confirm or delete items, rate meals on a 1- to 5-star scale, and add alternative foods or activities.

### Participants and Recruitment

Participants were recruited via hospital and newspaper advertisements, outreach to physicians, and online postings. Eligibility criteria are given in Textbox 1.

**Inclusion and exclusion criteria.**

Inclusion criteria

- Prediabetes or T2D (HbA_1c_ ≥5.7%, fasting glucose ≥100 mg/dL, or 2-hour glucose ≥140 mg/dL) [29]
- Age >18 years
- BMI 20-45 kg/m^2^

Exclusion criteria

- Type 1 diabetes
- Severe metabolic conditions
- Insulin, sulfonylureas, and glucagon-like peptide-1 (GLP-1) agonists
- No Android smartphone
- Use of continuous glucose monitoring (CGM) or activity tracking apps in the last 3 months

Some eligibility criteria were further specified during study implementation to ensure participant safety. In particular, individuals using sulfonylureas and glucagon-like peptide-1 receptor agonists were excluded, although these criteria were not explicitly detailed in the trial registry. The BMI inclusion range (20-45 kg/m^2^) was applied consistently during recruitment.

After telephone screening, 71 candidates were invited for on-site screening.

The PROTEIN trial conducted in Germany was retrospectively registered after completion of data collection at ClinicalTrials.gov: registration number NCT05951140 [30]; that said, the protocol and outcomes had been defined prior to data analysis.

### Meal Plan Personalization

Meal plans were developed using the PROTEIN knowledge tracker, tailored for prediabetes or T2D as shown in Table 1, and based on participant profile (age, sex, diagnosis, weight, dietary history, goals, allergies, intolerances, and religious preferences). Plans were updated biweekly, with participant feedback and CGM data guiding further personalization.

**Table 1:** Dietary framework of the knowledge tracker developed for prediabetes and type 2 diabetes.

Nutrient recommendations	Male	Female
Energy intake (kcal)	2100 (300)	2100 (300)
Salt (g)	<5	<5
CHO^a^ (%EI^b^)	45 (10)	45 (10)
Sugar (%EI)	<10	<10
Protein (g/kg/BW^c^)	0.8-1.4	0.8-1.4
Fat (%EI)	30 (10)	30 (10)
Fiber (g/day)	30-45	30-45
Fruit (portions)	2	2
Vegetables (portions)	≥3	≥3

^a^CHO: carbohydrates.

^b^EI: energy intake.

^c^BW: body weight.

Participants interacted with the app by confirming, deleting, or rating meals (1-5 stars) and activities. Customization increased as participants engaged with the app, especially during the initial 2 weeks, enabling the AI to learn user preferences. Engagement metrics were monitored to assess correlations with health behavior changes.

### Glucose Data

Each participant used a Freestyle Libre CGM system, with data uploaded weekly to inform recommendations. A TIR ≥70% (equivalent to HbA_1c_ ≤6.7%) was set as the target, following the international consensus on CGM [31].

If TIR dropped below 70%, the app automatically reduced daily carbohydrate recommendations by 10% for the following week. This allowed a stepwise personalization within the ranges of evidence-based dietary recommendations for the dietary management of diabetes [32,33]. Foods, apart from vegetables and pulses, causing high glucose excursions (≥140 mg/dL for 2-4 hours postprandial, on ≥2 occasions), activated a push notification directly to the user and were excluded from the meal plans the following week.

The data extracted from the CGM was only used if at least 70% of the glucose data was available per day. We based this modified approach on the recommendations for interpretations of TIR [31]. We compared the average TIR of the user during the time they were actively using the app to the average TIR during their planned waiting time in the study protocol. This approach was used to account for the fact that most participants did not use the application for the planned 12 weeks.

### Sample Size and Power Calculation

The study aimed for 300 participants to detect a 5% TIR difference with 80% power (β=0.20) and α=0.05, enabling detection of medium to small effects (Cohen *d*=0.348) [34]. Due to recruitment challenges, only 27 participants were enrolled, and 21 were included in the final analysis. The study is substantially underpowered for our primary aim, and the analysis that focused on periods of active app use should be interpreted as exploratory.

### Statistical Analysis

Initially, an as-treated analysis was planned for all completers. Due to low adherence and high dropout, the originally planned RCT analysis was not feasible. Therefore, the analyses presented are exploratory and were limited to periods of active app use compared with nonuse days. This approach introduces selection bias and does not preserve the randomized design. No intention-to-treat analysis was possible due to the limited sample size and missing data. Subgroup analyses (start-group vs wait-group) and analyses restricted to periods of active app use were not prespecified and should be interpreted as exploratory. Active app use days were defined as days with at least one logged interaction with the PROTEIN app and valid CGM data. Noncompliance was defined as <30% app use (<25 days) [35]. Statistical analyses were performed using SPSS 26.0 and Prism 8. Data are presented as mean (SD) in tables or mean (SEM) in figures. Paired 2-tailed *t* tests or nonparametric equivalents were used as appropriate, with significance set at *P*<.05. Outliers (>3× IQR) were excluded.

Due to early termination of recruitment, high attrition, and deviation from the planned randomized analysis, this study should be considered an exploratory pilot study. All analyses are hypothesis-generating and do not provide confirmatory evidence of effectiveness.

### Registered Outcomes and Reporting

To ensure transparency between the prespecified trial registration and the analyses reported in the results section, all registered outcomes were systematically compared with the outcomes analyzed. A summary of registered primary and secondary outcomes, their reporting status in this manuscript, and reasons for nonreporting where applicable, is provided in Table 2.

**Table 2:** Comparison of registered outcomes and outcomes reported in this manuscript.

Outcome (as registered at ClinicalTrials.gov)	Type	Reported in this manuscript	Reason for nonreporting (if applicable)
Time in range (TIR)	Primary	Yes	—^a^
Improve eating behavior toward healthier choices	Primary	No	Insufficient and inconsistent dietary data and absence of a predefined validated combined measure
Adherence to the dietary recommendations of the app	Secondary	Exploratory and descriptive only	Exploratory indicators (eg, app engagement, meal ratings) reported
Glycemic metabolism (HbA_1c_^b^, fasting glucose)	Secondary	Yes	—
Sedentary time	Secondary	No	Data are incomplete and not consistently recorded across participants
Energy intake	Secondary	Partially	Reported descriptively (Table 7); not analyzed as a formal outcome due to limited sample size
Microbiome	Secondary	No	Insufficient samples available for robust analysis
Polygenic risk score	Secondary	No	Not available for the analyzed cohort

^a^Not applicable.

^b^HbA_1c_: glycated hemoglobin A_1c_

In particular, the primary outcome “healthier eating behavior” could not be formally analyzed due to insufficient and inconsistent dietary data.

### Ethical Considerations

The pilot study PROTEIN was conducted at the University Hospital Charité. The ethics committee of the Charité University Hospital Berlin approved all trial procedures and amendments (approval number EA4/110/20 on September 11, 2020). We retrospectively registered the trial at ClinicalTrials.gov (registration number NCT05951140) after completion of data collection due to administrative delays; we defined the protocol and outcomes prior to data analysis. Written informed consent was obtained from all participants.

### CONSORT Guidelines

This randomized controlled trial was conducted in accordance with the CONSORT (Consolidated Standards of Reporting Trials) 2025 guidelines for reporting RCTs (Multimedia Appendix 1) [36].

## Results

### Demographic Characteristics

**Figure 2:**
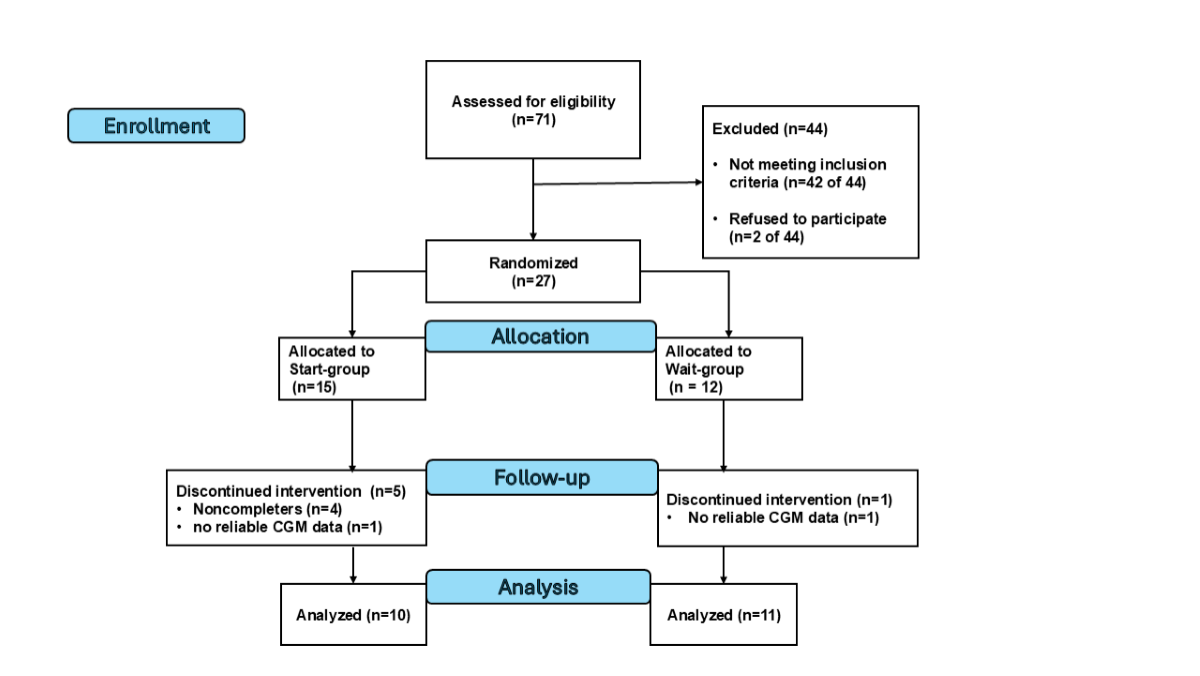
Flowchart depicting the progression of study participants throughout the pilot study. CGM: continuous glucose monitoring.

Overall, 27 participants (n=13 male, n=14 female) were enrolled in the study. Twelve participants (44%) stopped using the PROTEIN app after 12 days, 11 of whom continued to use the wearables. In total, 21 participants had reliable CGM data and were included in the analysis (12 female and 9 male) (Figure 2). The baseline characteristics of the participants are shown in Table 3.

**Table 3:** Baseline characteristics of the analyzed study participants.

Characteristics		Baseline (n=21)
Age (y), mean (SD)		57 (10.7)
Sex (female/male), n		12/9
Body composition, mean (SD)		
	Body weight (kg)	90.5 (19.2)
	BMI (kg/m^2^)	30.5 (6.6)
	Waist circumference (cm)	103.5 (12.5)
	Hip circumference (cm)	111.5 (14.8)
	WHR^a^	0.93 (0.08)
	Fat mass (kg)	26.5 (13.4)
	Fat mass (%)	28.4 (10.2)
Blood pressure (mmHg), mean (SD)		
	Systolic	137 (12)
	Diastolic	88 (8)
HbA_1c_^b^ (%)		6.4 (0.9)
Fasting blood glucose (mg/dl)		120.4 (34.3)
Liver transaminases (U/l), mean (SD)		
	ALT^c^ (GPT^d^)	28 (13)
	γ-GT	50 (65)
Lipid concentrations (mg/dl)		
	Total cholesterol	195 (54)
	Triglyceride	143 (87)
	LDL^e^-cholesterol	121 (47)
Prescribed oral antidiabetic medication		
	0	15
	1	2
	2	4

^a^WHR: waist to hip ratio.

^b^HbA_1c_: glycated hemoglobin A_1c_.

^c^ALT: Alanine aminotransferase.

^d^GPT: glutamate pyruvate alanine aminotransferase.

^e^LDL: low-density lipoprotein.

### CGM Data

We compared the TIR of the participants during the waiting phase (without the use of the PROTEIN app) to the intervention (with the use of the PROTEIN app). TIR increased significantly (*P*=.04), rising from 71.8% (SD 27.3%) to 76% (SD 28.1%). This represented a 4.2% (9%). Improvement, not meeting the 5% target set.

We then examined potential differences between the wait- and the start-group. In the start-group (n=10), the TIR decreased (not statistically signiﬁcant) from 68.9 (SD 32.7%) to 64.9 (SD 31.1%) (*P*=.14) once they stopped using the application, whereas the TIR of the wait-group (n=11) increased from 78.0 (SD 23%) to 82.4 (SD 22.9%) (*P*=.10) once they started using the application. Having considered the positive effect on the TIR, we investigated whether starting to use the PROTEIN app first (start: 4, SD 9.4 vs wait: 4.3, SD 8) yielded a stronger change in the TIR. No significant difference in the change of the TIR between the 2 groups was seen (*P*=.94).

### Routine Glucose Parameters

HbA_1c_ and fasting blood glucose did not change significantly during the study, as shown in Tables 4 and 5. Values remained comparable across study visits within both groups. No statistically significant differences were observed between the intervention and waiting phases.

**Table 4:** Glycated hemoglobin A1c separated by group and study phase.

Group phase^a^		Visit	Participants, n	HbA_1c_^b^ (%), mean (SD)	*P* value				

Start group									
	Intervention					.11			
		V1	8	6.8 (1)					
		V2	8	6.5 (0.6)					
	Waiting					.47			
		V2	5	6.3 (0.7)					
		V3	5	6.4 (0.9)					
	Both					.22			
		V1	6	6.8 (1.1)					
		V3	6	6.6 (0.9)					
Wait group									
	Waiting					.26			
		V1	10	6.2 (0.9)					
		V2	10	6.1 (0.8)					
	Intervention					.36			
		V2	4	6 (0.7)					
		V3	4	6.1 (0.6)					
	Both					.41			
		V1	4	6 (0.6)					
		V3	4	6.1 (0.6)					

^a^HbA_1c_ changes during the different phases (intervention and waiting) of the pilot. “Intervention” represents the period of time where the participants use the app; “Waiting” represents the waiting phase without the use of the app; and “both” represents both phases together. P represents *P* values from *t* test for analyzing differences within each group.

^b^HbA^1c^: glycated hemoglobin A_1c_.

**Table 5:** Fasting blood glucose separated by group and study phase.

Group phase^a^		Visit	Participants, n	Fasting blood glucose (mg/dL), Mean (SD)	*P* value	
Start group						
	Intervention					.31
		V1	7	134.7 (41.8)		
		V2	7	122.9 (24.7)		
	Waiting					.50
		V2	5	115.4 (26.1)		
		V3	5	125.2 (48.2)		
	Both					.72
		V1	5	128.8 (39.2)		
		V3	5	131.6 (48.8)		
Wait group						
	Waiting					.72
		V1	10	109.5 (29.8)		
		V2	10	104.5 (19.7)		
	Intervention					.07
		V2	4	101.3 (17.3)		
		V3	4	107.5 (17.7)		
	Both					.07
		V1	4	101.3 (17.5)		
		V3	4	107.5 (17.7)		

### Body Weight Change

We analyzed the wait-group and start-group separately. Body weight remained stable within and between the wait-group and start-group as shown in Table 6. Mean values were similar across study visits in both groups. No statistically significant changes were observed over time.

**Table 6:** Body weight separated by group and study phase.

Group phase^a^		Visit	Participants, n	Weight (kg), mean (SD)	*P* value		
Start group							
	Intervention					.92	
		V1	8	96.5 (18)			
		V2	8	96.6 (17.3)			
	Waiting					.14	
		V2	5	95.7 (18.9)			
		V3	5	94.9 (19.3)			
	Both					.11	
		V1	6	93.5 (21.7)			
		V3	6	91 (19.7)			
Wait group							
	Waiting					.65	
		V1	11	87.3 (20.3)			
		V2	11	86.8 (21.3)			
	Intervention					.24	
		V2	5	91.2 (26.7)			
		V3	5	92 (26.7)			
	Both					.87	
		V1	5	92.1 (25.9)			
		V3	5	92 (26.7)			

### Use of the PROTEIN App

The intended duration of the PROTEIN app usage was 84 days (12 weeks), but only 2 participants completed the full period. To account for this, we assessed the time period between first and last use of the PROTEIN app, irrespective of intermittent pauses. Participants’ average app usage was 42 days (range 5-84 days). Contrary to expectations, there was no significant correlation between days of app usage and change in TIR (r= –0.224; *P*=.33). No adverse events or study-related harms were observed during the study period.

### Personalization

The process of personalization is described in detail in the PROTEIN AI-Advisor [8].

To test the accuracy of the recommended meal plans, the data from the nutrition and activity plans were extracted and compared to the knowledge tracker, as seen in Table 7. We did not detect any high glucose excursions caused by pulses or vegetables, nor exclusions of specific foods.

**Table 7:** Nutrient recommendations via the PROTEIN app compared to the knowledge tracker.

Nutrient recommendations	Values, mean (SD; n=21)	Knowledge tracker (male/female)
Energy intake (kcal)	2048 (376)	2100 (300)/1750 (250)
Protein (g/kg/BW)	1.2 (0.2)	0.8-1.4
Carbohydrates (%EI)	49 (5)	45 (10)
Fat (%EI)	31 (4)	30 (10)
Fiber (g/d)	36 (6)	30-45
Fruit (portions/day)	3 (1)	2
Vegetables (portions/day)	5 (2)	≥3

Overall, the average star ratings ranged from 3 out of 5 stars for lunch and dinner to 3,5 out of 5 stars for breakfast, afternoon snack, and supper, and 4 out of 5 stars for morning snack.

In addition, we investigated the total variety of meal composition that was yielded for 17 participants; 159 unique options for breakfast were recommended, 114 for morning snacks, 145 for lunches, 112 for afternoon snacks, 123 for dinner, and 27 for suppers. Furthermore, 6 (29%) participants reported trying new meals that they had not thought of before as a result of using the PROTEIN app.

## Discussion

Our study addressed two research questions: whether the PROTEIN app increases TIR and whether it affects body weight. The results indicate a modest improvement in TIR (4.2%) during app use, with no change in weight. In summary, our data indicated that the TIR improved with the use of the PROTEIN app for only an average of 12 days, but this fell short of the expected 5% improvement and of the expected 12-week usage period. Unexpectedly, we did not find a significant correlation between the duration of app use and the improvement of the TIR, which could be due to the small sample size and narrow range of usage durations. Despite the high rates of noncompliance in the intervention phase, the TIR improved in parallel with the use of the mobile nutrition app, suggesting a potential signal that a tailored recommendation approach delivered with wearables through a mobile app may be associated with small increases in TIR and may encourage greater concordance with dietary recommendations. However, this observation is exploratory and cannot be distinguished from selection bias or confounding, and any effect on dietary concordance remains uncertain. Our study, therefore, also illustrates the need to balance technological capabilities with user experience since using an app with many features and wearables is time-consuming, contributing to participant fatigue and dropout.

Overall, participants who took part in the intervention with valid CGM data (n=21) had an improvement of 4.2% in their TIR while they were using the PROTEIN app, suggesting that even though they did not follow the recommendations every day, there seems to be a degree of agreement and engagement while using the PROTEIN system [35,37]. It is noteworthy that the outcome relies on the adequate resources of the participants and may be influenced by different factors, including family network, profession, and socio-economic status.

Although the 4.2% increase in TIR reached statistical significance, its clinical relevance remains uncertain, because of the high baseline TIR (mean >70%) and the lack of change in HbA_1c_, fasting glucose, and weight. International consensus recommends achieving and maintaining TIR ≥70% and states that a 5% change in TIR is strongly associated with a 0.5% decrease in HbA_1c_, which is a clinically significant reduction. We therefore interpret the observed TIR change as a modest signal of potential benefit that requires confirmation in larger, adequately powered studies.

We anticipated that participants would use the PROTEIN app for a full 12 weeks; however, this was not achieved by the majority. This was largely due to the app being perceived as not user-friendly and occasionally too time-consuming. Defining dropouts was a difficult task, since several studies defined this differently. We based our definition of adherence on the days the app was used, namely, at least 30% of the planned time. According to this definition, the dropout rate in the study (43%) is consistent with the work reported by Meyerowitz-Katz et al [38].

Accordingly, after analyzing the differences between the two groups, we observed that the participants’ TIRs improved regardless of whether they started with the intervention or the waiting phase. This may suggest that wearables alone are not sufficient to improve glucose management, but that extra feedback, in this case in the shape of an app, led participants toward better glucose management, although this cannot be determined definitively from the current data. The TIR of the start-group did not decrease significantly after they stopped using the PROTEIN app, suggesting there is a legacy effect for the users. The participants kept using the sensors as planned (12 weeks during intervention and 6 weeks in the waiting phase), which allowed us to compare the two periods of time. From these data, we observed patterns consistent with improved glucose management during the PROTEIN app use; however, causality cannot be inferred due to the exploratory design and potential selection bias. A dietary protocol before intervention could be added in further research to provide a better understanding of dietary change.

Furthermore, prior studies have shown that CGM is an important tool for the management of T2D [13,39,40]. When we analyzed our participants according to the assigned group, we can see that using wearables without guidance on how to effectively use them did not cause a change in the glucose levels. Interestingly, the participants who used the PROTEIN app right away (start-group) had a decrease in the TIR once they stopped using it, but this decrease was not statistically significant. This suggests that 12 weeks may not be enough to maintain a health-supporting behavior for a longer period. As no significant changes in weight were seen over the study period, improvements in TIR appear to be driven by the consumption of more adequate meals, independent of the group they were randomized into. Therefore, we cannot determine whether the PROTEIN app influences their caloric intake, but the findings are consistent with changes in food selection.

Overall, the PROTEIN application generally provided suitable meals for individuals with T2D and prediabetes. However, on a few occasions, the recommended protein amounts fell short when compared to the knowledge tracker. The first 2 weeks during which the participants were expected to train the app proved to be excessively time-consuming, leading to a decline in the compliance of our participants. Additional factors, including managing multiple devices and integrating the protocol into daily routines, were also identified as reasons for disengagement. The mobile app was programmed to change the meal plan for the whole week once the subject skipped a meal and added a new one instead. Moreover, the PROTEIN app sometimes failed to “learn” or adapt after two weeks, despite participants having the same breakfast daily.

The PROTEIN AI-advisor adjusted carbohydrate targets in response to low TIR and was programmed to exclude foods associated with repeated high excursions. We could not detect the frequency and magnitude of these adaptations in practice. Moreover, participants reported cases in which the system did not sufficiently adapt to repeated meal patterns, indicating that the current personalization was only partially realized and requires further optimization.

It is important to acknowledge that 21 participants had reliable CGM data, making the trial substantially underpowered and exploratory relative to the original sample size calculation with 300 participants. All suggestions of effect should be interpreted as exploratory signals rather than solid evidence.

Furthermore, limiting analyses to periods of active app use introduces substantial selection bias toward more engaged users, likely underestimates variability in real‑world use, and may overestimate potential effects.

Several limitations apply to our exploratory pilot study. Recruitment and retention were challenging, exacerbated by the COVID-19 pandemic and technical barriers (eg, Android-only compatibility). These factors limited the sample size and statistical power, highlighting the need for larger, more inclusive studies. Several prespecified outcomes were registered in the clinical trial registry but are not reported in this manuscript because of the limited sample size and incomplete data collection. A detailed comparison of registered and reported outcomes is provided in Table 2. Even though we saw a moderate difference in the TIRs while using the PROTEIN app, we were unable to assess participants’ TIR prior to using the wearables and/ or using the application, which could provide further robust evidence as to the health behavior changes.

Therefore, we cannot fully exclude the effects influencing intra-participant TIR patterns, as preintervention blind CGM data periods were not available.

Despite these limitations, the study provides preliminary indications of the feasibility and potential of combining mHealth, AI, and CGM data to support individualized diabetes care; nevertheless, it must be recognized that our own evidence indicates the intervention does not feasibly support increased TIR, except for a short period and for a small proportion of app users. This emphasizes important limitations regarding the broader clinical use and sustainability of the approach.

Our exploratory pilot study adds novel data on the feasibility, adherence patterns, and preliminary glycemic impact of such an AI-driven system under real-world conditions.

Future research should focus on improving user engagement, expanding compatibility, and evaluating long-term clinical outcomes in larger, more diverse populations.

### Conclusions

The level of personalization offered by the PROTEIN app needs to be enhanced [9], which could help reduce the time required for the app to learn and adapt. For example, allowing users to select in advance their favorite ingredients or meals could streamline the learning process. Ideally, greater integration of image recognition features, allowing participants to take and upload pictures of their meals rather than manually entering ingredients, and the ability to automatically upload and correlate CGM data with food intake would significantly improve the user and researcher experience. These conclusions are based on this combination of published evidence and participant input.

Considering the strong limitation in sample size and engagement relative to the original protocol, our study results should be seen as an exploratory trial that primarily provides information regarding feasibility, engagement, and potential effects.

Finally, the PROTEIN app was associated with a modest increase in TIR in adults with prediabetes or T2D; however, this exploratory finding does not establish effectiveness and requires confirmation in adequately powered trials, suggesting a possible role for AI-driven, personalized mHealth solutions for diabetes management. While the primary endpoint was not fully achieved, the study supports further development and evaluation of adaptive digital interventions designed to enhance personalized engagement with dietary recommendations and improve glycemic control.

## Data Availability

The data that support the findings of this study, trial protocol, and statistical analysis are not publicly available to preserve individuals’ privacy under the European General Data Protection Regulation but are available from the corresponding author (elena.lalama@charite.de) upon reasonable request.

## Appendix

Multimedia Appendix 1 CONSORT checklist.

## Abbreviations

AI: artificial intelligence
CGM: continuous glucose monitoring
HbA1c: glycated hemoglobin A1c
mHealth: mobile health
PA: physical activity
PDA: personal digital assistants
PROTEIN: personalized nutrition for healthy living
RCT: randomized controlled trial
T2D: type 2 diabetes
TIR: time in range
WHO: World Health Organization
